# Early-phase factors associated with pediatric severe dengue in the Thai–Myanmar cross-border region

**DOI:** 10.1186/s12889-024-19492-9

**Published:** 2024-07-22

**Authors:** May Thu Thu Aung, Noppadon Tangpukdee, Kriengsak Limkittikul, Ramparat Keeratiwasin, Rungrat Sukharom, Weerawan Hattasingh, Salin Sirinam

**Affiliations:** 1 Department of Tropical Pediatrics, Faculty of Tropical Medicine, Mahidol University, Bangkok, Thailand; 2 Department of Clinical Tropical Medicine, Faculty of Tropical Medicine, Mahidol University, Bangkok, Thailand; 3 Department of Pediatrics, Maesot General Hospital, Tak, Thailand

**Keywords:** Dengue infection, Severe dengue, Children, Cross-border

## Abstract

**Background:**

Dengue disease is caused by dengue virus, which is transmitted by *Aedes* mosquitoes in tropical and subtropical regions worldwide. Although most infected individuals have benign febrile illness or no apparent symptoms, a small percentage develop severe dengue, a potentially fatal condition that occurs after a febrile stage. Many studies have identified factors predicting dengue severity among different populations and time courses. To help find practical approaches applicable in remote settings, we focused on the investigation of early factors associated with severe dengue in Thai–Myanmar cross-border region.

**Methods:**

This retrospective case-control study was performed to determine factors contributing to severe dengue in the pediatric population. We reviewed the hospital records of patients with dengue infection aged 0–19 years who were admitted to Maesot General Hospital, situated near the Thai–Myanmar cross-border region, between 2017 and 2022. Medical data during the first 5 days of illness and outcomes were collected and analyzed.

**Results:**

This study included 144 patients with a serologically confirmed diagnosis of dengue infection, with 43 severe and 101 non-severe cases. Among biological factors, being an infant and belonging to an ethnic group in Myanmar showed a significant association with severe dengue in the univariable analysis. Multivariable logistic regression revealed that the presence of mucosal bleeding (adjusted OR 5.39, 95% CI 1.06–27.52, *P* = 0.043), a change in hematocrit ≥ 10% (adjusted OR 3.68, 95% CI 1.15–11.74, *P* = 0.028), and serum albumin < 35 g/L (adjusted OR 8.10, 95% CI 2.55–25.72, *P* < 0.001) during the first 5 days of illness were significantly associated with developing severe dengue.

**Conclusions:**

This study supports the use of certain WHO warning signs and hematocrit change during febrile phase to predict pediatric severe dengue in low-resource settings. Potential factors such as very young age and ethnic groups warrant further exploration to identify risks contributing to severe dengue infection.

## Introduction

Dengue disease is caused by dengue virus, a positive single-stranded RNA virus that is a member of the *Flaviviridae* family. *Aedes* mosquitoes, which are primarily found in tropical and subtropical regions of the world, transmit the disease. The incidence of dengue has increased substantially worldwide, with estimates that nearly half the world’s population is at risk [1, 2]. The incubation period for dengue virus is 4–10 days after a bite from an *Aedes* mosquito, and 80–90% of those infected experience no symptoms. However, according to the 1997 World Health Organization (WHO) classification [3], 10–20% of affected individuals exhibit clinical symptoms, with approximately 50% showing moderate symptoms, and 40% developing dengue fever. In approximately 10% of infected individuals, more severe manifestations are seen, which are classified as dengue hemorrhagic fever; among these patients, at least 5% develop dengue shock syndrome. In 2009, a new dengue case definition was published, categorizing infection into dengue with or without warning signs and non-severe dengue or severe dengue [4]. Previous studies have shown that the revised 2009 classification is more sensitive for anticipating disease severity than the 1997 WHO dengue guidelines and more convenient for use in triage during outbreaks and in the management of dengue [5].

Numerous studies have identified the risk factors associated with severe dengue in pediatric populations across diverse regions and stages of illness. Severe dengue, which includes severe plasma leakage, severe bleeding, and severe organ dysfunction such as acute liver failure and acute renal injury, is the primary cause of hospital admissions and deaths in affected pediatric patients [6]. Despite the fact that death owing to dengue occurs in a small percentage of cases, once shock sets in, this rate can rise to 12–44%. The cross-border Thai–Myanmar region is an endemic area for vector-borne diseases including dengue. This region has unique patient as well as public health characteristics. Several articles have described the challenge of health care accessibility in this area. In cases of acute febrile illness occurring within 1–3 days, patients on the Thai-Myanmar border mostly self-medicate. If the fever persists, they begin seeking various medical services along the border, including Thai public hospitals, which increases the strain on these facilities [7–9]. Healthcare providers also face challenges in managing infectious diseases at the border, such as shortage of hospital staffs and capacities, referral system issue, and treatment plan failure. The socioeconomic status of patients is one of the key determinants affecting both patients and healthcare providers. Distance from medical units, legal status, and cultural differences were found to be associated with the health-related behavior of the patients. Some patients only reached healthcare services when the symptoms advanced or they were unable to perform daily activities. These barriers also pose challenges for resource management by healthcare providers [7, 10]. Therefore, to improve case management and lower the cost of dengue-related medical treatment, early detection of potentially severe illness is essential. In this study, we aimed to identify early factors associated with severe dengue in pediatric patients admitted to a tertiary hospital in the Thai–Myanmar border region.

## Methods

### Study design and location

Maesot General Hospital is a standard-level referral hospital located in the Thai-Myanmar border area of Tak Province, Thailand. Situated 500 km from Bangkok, the capital city of Thailand, it is only 6 km from the Thai–Myanmar border. Maesot General Hospital is the largest hospital in Tak Province’s five westernmost districts and serves a registered population of approximately 250,000 people, of which half are non-Thai belonging to diverse ethnic groups. However, owing to the geographic location of the border, this hospital cares for a considerably large number of unregistered patients. The hospital can provide efficient management and intensive care treatment for severe dengue and thus had relevant data available for this study. The medical records of hospitalized patients from 2017 to 2022 were reviewed to identify risk factors for pediatric severe dengue in the border area of Maesot. The inclusion criteria were patients aged 0–19 years and a clinical diagnosis of dengue infection confirmed by either positive polymerase chain reaction, non-structural protein 1 antigen (NS1 Ag), or immunoglobulin (Ig) M tests. Patients with incomplete medical records, including a lack of properly recorded physical signs or incomplete clinical data progression, were excluded.

The medical records of patients hospitalized with dengue from 2017 to 2022 who met the inclusion and exclusion criteria were used retrospectively in this study. Data were collected using a case record form that included patient details such as age, sex, race and ethnicity, and underlying medical conditions. The nutritional status of patients was categorized using the body mass index chart for Thai children as reference [11]. Clinical symptoms appearing during the first 5 days of illness included the day of fever onset and the presence or absence of warning signs, as defined in the WHO criteria [4]. Laboratory results during the first 5 days of illness, as well as dengue serology (NS1 Ag, IgM, and IgG) and treatment administered, were collected as variables for the analysis. The outcome of severe and non-severe dengue was based on the 2009 WHO classification [4].

This study was approved by the Ethics Committee of the Faculty of Tropical Medicine, Mahidol University, under protocol TMEC 23 − 012, and the Human Research Ethics Committee of Maesot General Hospital, Ministry of Public Health, Thailand.

### Statistical analysis

We performed the data analysis using SPSS version 18 (SPSS Inc., Chicago, IL, USA). In the analysis, we used descriptive statistical including frequency distribution, proportion, mean, and standard deviation. To assess the association of risk factors for severe dengue, univariable and multivariable logistic regression analysis were used. Statistical significance was set at a two-tailed 95% confidence level (*P* < 0.05).

## Results

According to the inclusion and exclusion criteria, 144 patients were finally enrolled in the analysis, including 43 with severe dengue (only 1 death) and 101 with non-severe dengue. In detail, the classification of severe dengue based on WHO criteria 2009 is described using three categories: severe plasma leakage, severe bleeding, and severe organ involvement [4]. In the severe group, 32 individuals had severe plasma leakage, followed by 22 events of severe bleeding. Severe organ involvement was detected in 11 patients with severe infection, mostly severe transaminitis, followed by acute kidney injury. Five patients with dengue had co-infections: one with scrub typhus, two with COVID-19 infection, and two with severe *Plasmodium falciparum* and *P. vivax* infection. The characteristics of participants and their distributions are shown in Table 1. Belonging to an ethnic group from Myanmar and infant aged < 1 year (compared with adolescent age 13‒19 years) had higher odd ratios (ORs) for severe dengue (OR 4.52, 95% confidence interval [CI] 2.08‒9.85 and OR 3.41, 95% CI 1.06‒11.03, respectively). Sex, nutritional status, underlying diseases, co-infection, and a pattern of serostatus positivity did not show significant associations with dengue severity.

**Table 1:** Characteristics of patients and association with dengue severity

Characteristics (*n* = 144)	Number (%)	Severe dengue (*n* = 43)	Non-severe dengue (*n* = 101)	Crude odds ratio (95% CI)	*P*
**Sex**					
Male	71 (49.3)	19 (44.2)	52 (51.5)	1	
Female	73 (50.7)	24 (55.8)	49 (48.5)	1.34 (0.65‒2.75)	0.423
Ethnicity*					
Thai	103 (71.5)	21 (48.8)	82 (81.2)	1	
Myanmar	41 (28.5)	22 (51.2)	19 (18.8)	4.52 (2.08‒9.85)	< 0.001
Age in group*					
Infant < 1 year*	15 (10.4)	9 (20.9)	6 (5.9)	3.41 (1.06‒11.03)	0.040
1‒5 years	13 (9.0)	1 (2.3)	12 (11.9)	0.19 (0.02‒1.57)	0.123
6‒12 years	57 (39.6)	15 (34.9)	42 (41.6)	0.81 (0.36‒1.83)	0.617
13‒19 years	59 (41.0)	18 (41.9)	41 (40.6)	1	
**Nutritional status (** *n* ** = 107)**					
Normal	79 (73.8)	18/23 (78.3)	61/84 (72.6)	1	
Underweight	8 (7.5)	3/23 (13.0)	5/84 (6.0)	2.03 (0.44‒9.34)	0.362
Overweight	14 (13.1)	1/23 (4.3)	13/84 (15.5)	0.26 (0.03‒2.13)	0.210
Obesity	6 (5.6)	1/23 (4.3)	5/84 (6.0)	0.68 (0.07‒6.18)	0.730
**Underlying diseases**					
No	137 (95.1)	40 (93.0)	97 (96.0)	1	
Yes	7 (4.9)	3 (7.0)	4 (4.0)	0.37 (0.04‒3.14)	0.675
**Co-infection**					
No	139 (9)	40 (93.0)	99 (98.0)	1	
Yes	5 (6.3)	3 (7.0)	2 (2.0)	1.97 (0.50‒7.77)	0.452

*Values statistically significant at *P* < 0.05.

CI, confidence interval.

Clinical manifestations in patients with dengue were evaluated to assess the association with dengue severity. We assessed the duration of fever and clinical symptoms based on the WHO warning signs criteria [4]. The average duration of fever was statistically different between the severe dengue group and non-severe dengue group (7.16 ± 4.98 days vs. 5.04 ± 2.03 days, respectively; *P* < 0.001). A duration of fever longer than 5 days was more likely to result in greater severity (OR 3.48, 95% CI 1.65‒7.32). Warning signs of liver enlargement, lethargy/restlessness, and mucosal bleeding showed statistical significance (OR 8.03, 95% CI 1.55–41.56; OR 3.94, 95% CI 1.57–9.91; and OR 3.62, 95% CI 1.17–11.17, respectively) in the severe group, as compared with the non-severe group. Patients presenting with additional warning signs of persistent vomiting and abdominal pain had a greater likelihood of having severe dengue infection, but this was not statistically significant. Additionally, dengue symptoms that were not defined as warning signs did not show statistical significance in a comparison between severe and non-severe patients. As for laboratory markers, we investigated whether deviated cut-off values were associated with dengue severity. Patients who had a relative increase or decrease in hematocrit level of ≥ 10% during the first 5 days of illness, white blood cell count ≥ 4 × 10^9^/L, platelet count < 100 × 10^9^/L, and serum albumin < 35 g/L had higher odds of severe infection. Nevertheless, having IgG positive serology or triple positivity (positive NS1Ag, IgM, and IgG) were not associated with severe dengue infection (Table 2).

**Table 2:** Clinical signs and laboratory markers during the first 5 days of illness and dengue severity

Number, %	Severe Dengue (*n* = 43)	Non-severe Dengue (*n* = 101)	Crude Odds Ratio (95% CI)	*P*
Duration of fever*				
1‒5 days	16 (37.2)	68 (67.3)	1	0.001
>5 days	27 (62.8)	33 (32.7)	3.48 (1.65‒7.32)	
**Dengue warning signs**				
Lethargy/restlessness*	13 (30.2)	10 (9.9)	3.94 (1.57‒9.91)	0.002
Persistent vomiting	13 (30.2)	21 (20.8)	1.65 (0.72‒3.71)	0.222
Abdominal pain	10 (23.3)	21 (20.8)	1.15 (0.49‒2.72)	0.742
Liver enlargement*	6 (14.0)	2 (2.0)	8.03 (1.55–41.56)	0.009
Mucosal bleeding*	8 (18.6)	6 (5.9)	3.62 (1.17‒11.17)	0.019
Fluid accumulation	8 (18.6)	0 (0.0)	N/A	N/A
**Dengue symptoms**				
Headache	11 (25.6)	30 (29.7)	0.81 (0.36‒1.82)	0.616
Myalgia	9 (20.9)	24 (23.8)	0.85 (0.36‒2.02)	0.711
Rash	4 (9.3)	8 (7.9)	1.19 (0.34‒4.19)	0.751
Respiratory symptoms	15 (34.9)	33 (32.7)	1.104 (0.52‒2.34)	0.797
Diarrhea	4 (9.3)	23 (22.8)	0.35 (0.11‒1.08)	0.065
Petechiae	8 (18.6)	14 (13.9)	1.42 (0.55‒3.68)	0.469
**Laboratory markers**				
Hematocrit difference ≥ 10%*	26 (60.4)	35 (34.6)	2.84 (1.36‒5.93)	0.005
WBC count < 4 × 10^9^/L*	17 (39.5)	60 (59.4)	0.45 (0.22‒0.93)	0.030
Platelets < 100 × 10^9^/L*	35 (81.4)	53 (52.5)	3.96 (1.67‒9.38)	0.002
Albumin < 35 g/L*	25 (58.1)	13 (12.9)	8.65 (3.57–20.98)	< 0.001
IgG positivity	28 (65.1)	56 (55.4)	1.58 (0.72‒3.50)	0.255
Triple positivity (NS1Ag, IgM and IgG dengue)	5 (11.6)	11 (10.9)	1.09 (0.33‒3.60)	0.896

*Values statistically significant at *P* < 0.05.

N/A, not applicable; CI, confidence interval; WBC, white blood cell; IgG, immunoglobulin G; NS1 Ag, non-structural protein 1 antigen.

Logistic regression analysis was performed to investigate associated factors during the early phase of severe dengue. Potential variables, as described previously, were included in the analysis. The results of multivariable analysis for biological factors, clinical signs, and laboratory markers are shown in Table 3. Infants aged less than 1 year had greater odds developing severe dengue compared with older children; however, only the group aged 1‒5 years showed statistical significance. Mucosal bleeding was the only warning sign associated with severe dengue (OR 5.39, 95% CI 1.06‒27.52). For laboratory parameters measured during the early phase, patients with a relative change (increase or decrease) in hematocrit level ≥ 10% and serum albumin < 35 g/L had greater odds of developing severe dengue (OR 3.68, 95% CI 1.15‒11.74 and OR 8.10, 95% CI 2.55‒25.72, respectively).

**Table 3:** Multivariable analysis of biological factors, clinical signs, and laboratory markers based on dengue severity

Factors	Adjusted Odds Ratio	95% CI	*P*
**Biological factors**			
Age groups*			
< 1 year 1‒5 years 6‒12 years 13‒19 years	1 0.01 0.26 0.44	0.00‒0.18 0.04‒1.70 0.07‒2.77	0.004 0.158 0.380
Ethnicity			
Thai Myanmar	1 2.53	0.80‒8.01	0.114
**Clinical signs**			
Lethargy/restlessness	3.16	0.74‒13.45	0.119
Liver enlargement	2.38	0.27‒20.66	0.432
Mucosal bleeding*	5.39	1.06‒27.52	0.043
**Laboratory markers**			
Hematocrit difference ≥ 10%*	3.68	1.15‒11.74	0.028
WBC count < 4 × 10^9^/L	0.49	0.16‒1.45	0.195
Platelets < 100 × 10^9^/L	1.01	0.28‒3.68	0.986
Albumin < 35 g/L*	8.10	2.55‒25.72	< 0.001

*Values statistically significant at *P* < 0.05.

CI, confidence interval; WBC, white blood cell.

## Discussion

The clinical spectrum of dengue infection varies from asymptomatic to severe forms, which can rapidly progress to life-threatening conditions. The WHO published a revised classification that categorizes dengue as severe or non-severe dengue, with or without warning signs. Identifying associated factors for severe dengue is crucial for timely intervention and preventing adverse outcomes [12].

In this study, we aimed to identify factors associated with pediatric severe dengue infection in the cross-border area of Thailand-Myanmar. Therefore, Maesot General Hospital in Tak Province, Thailand, was selected as it serves a large multi-ethnic population. Border health is one of the critical issues for public health. Previous studies have investigated the problems of infectious diseases in border areas [7–10]. As dengue is one of the major concerns among pediatricians in this region, we conducted the study specifically on dengue patients, which included the multi-ethnic characteristics of our study population. In the univariable analysis, the infant group showed an association with higher odds of severe dengue. However, patients older than age 5 years did not demonstrate a statistically significant difference compared with infants in the multivariate analysis. Previous studies have compared the severity of dengue among infants, children, and adults. Infants have a higher mortality rate and more dengue complications such as shock, severe leakage, marked thrombocytopenia, and fluid overload [13, 14]. One study found that approximately half of infants hospitalized with dengue subsequently developed severe disease [15]. Dengue antibodies acquired from the mother predispose infants to antibody-dependent enhancement (ADE) and severe clinical manifestations [16]. It is worth mentioning that patients from Myanmar had significantly higher odds for severe dengue compared with Thai patients in the univariable analysis, but this association did not persist in multivariable logistic regression. Although genetic factors may play a role in the severity of a particular disease [17], these are more likely to be associated with sociocultural factors, for example, limited health care access or the perception of illness, which have been debated in the context of other infectious diseases [7, 8, 18]. Additionally, socioeconomic factors also play an important role in border health. However, due to the retrospective nature of our study, we could not adequately explore these data in depth. Instead, our analysis indicated that ethnicity may also be associated with dengue severity. This finding could contribute to further prospective investigations to explore these association more comprehensively.

The existing warning signs defined in the WHO guideline for predicting severe dengue have been useful in clinical settings. Yet, the practical application of them can be challenging due to the lack of precise definitions for some symptoms, and its usefulness has still been investigated recently [5,19─^21^]. Despite these challenges, warning signs remain a simple tool for risk identification, allowing caregivers and healthcare providers to prioritize patients who may need more intensive care. By including these signs in the analysis of our population, we determined that lethargy/restlessness, liver enlargement, and mucosal bleeding were more likely to be associated with severe dengue than persistent vomiting and abdominal pain in this context. Previous publications have also found that lethargy and hepatomegaly were associated with severe dengue as compared with non-severe dengue [22]. For low-resource settings such as in cross-border areas, defining the warning signs for disease progression will benefit patients and families in terms of seeking health care.

In this study, basic laboratory markers also showed associations with severe dengue. During the first 5 days of illness, a relative increase or decrease in hematocrit level of ≥ 10%, which indirectly reflects a change in hemodynamics, was associated with severe infection. Numerous studies have investigated the impact of abnormal hematocrit on the risk of severe dengue. Both low and high hematocrit were found to be associated with an increased probability of severe dengue [20, 23–24]. Tantracheewathorn et al. found that a hemoconcentration > 22% from baseline was associated with dengue shock syndrome, which is in agreement with the WHO criteria of a dengue hemorrhagic fever cut-off value of 20% [23]. However, Phakhounthong et al. subsequently found that children with a hematocrit cut-off value of ≤ 28% had greater odds of severe dengue infection [20]. We found that a change in both directions of hematocrit of at least 10% during the early phase of the disease was linked to higher odds of developing severe dengue in children. Serum albumin has also been investigated in various studies, in both adult and pediatric populations, and is proven to be a critical factor predicting severe dengue [15, 21, 25]. Our study findings also confirmed this result and highlighted that an abnormal albumin level below 35 g/L in the first 5 days of illness increased the probability of severe infection.

Overall, this study has explored the risk of developing severe dengue in the vulnerable population living in the neglected area. The results showed that very young age and belonging to an ethnic group may be associated with severe disease. Specific warning signs of lethargy, liver enlargement, and mucosal bleeding may be the significant predictors and can be used practically in low-resource settings. In facilities where laboratory investigations can be performed, a complete blood count, particularly hematocrit levels remain a crucial test for risk stratification. A change in hematocrit level signifies hemodynamic change, and a relative change of at least 10% in dengue patients should be monitored. Serum albumin is an important chemistry test that could be performed in the higher centers to predict disease severity. Given the multi-faceted landscape of border health contributing to the factors of infectious disease, our data should lay the groundwork for more extensive investigations in the future to improve patient management and outcomes.

Our study might present with several limitations. The first was typical nature of retrospective study. Some details of patient histories and laboratory parameters were lacking. The second limitation was limited numbers of cases in the study due to a strict dengue diagnosis criterion, which relied on only serological tests and excluded a large number of clinically diagnosed cases. The third limitation was the exploratory nature of the analysis. The associated factors in our multivariate model are, at best, suggestive for further analysis with a higher number of cases that could provide a more statistically robust result [26]. Based on these limitations, subsequent studies should increase the number of cases through a multicenter study which could enhance the availability of cases, the completion of obtained medical records, and the power of statistical model.

## Conclusion

Some WHO warning sign criteria for dengue can support the early prediction of severe dengue in low-resource settings. Awareness should be raised among health care personnel that bedside hematocrit assessed during the early phase of infection can help to detect potentially severe dengue cases and prioritize management. Further investigation into the factors influencing dengue prognosis in cross-border regions is warranted. Considering a broader spectrum of factors, including vectors as well as environmental, sociocultural, and economic aspects, will improve understanding of the health care landscape in the study area.

## Data Availability

The clinical data set of this study are available upon reasonable request under the regulation of the Ethics Committee of the Faculty of Tropical Medicine, Mahidol University and Maesot General Hospital, Ministry of Public Health, Thailand.

## Abbreviations

g/L: gram per liter
NS1: non-structural protein 1 antigen
IgM: immunoglobulin M
IgG: immunoglobulin G
WBC: white blood cell
WHO: World Health Organization
